# The Treatment of Pneumonia

**Published:** 1905-01-28

**Authors:** 


### THE TREATMENT OF PNEUMONIA.

IN these sceptical times it is a pleasure to hear a physician speak with conviction of the measures to be used in treating a case of illness, and especially so when he is dealing with pneumonia, in which the therapeutic ineptitude of to-day is only too often displayed in the decrepit phrase "expectant treatment." Dr. Ewart[1] denounces with vigour the tendency to therapeutic inactivity. The physician, he thinks, has much to do in superintending a case of pneumonia, and must commence his treatment at the earliest moment. The first thing to be done is to meet the shock of the invasion by warmth and a small dose of ether or brandy with hot water, followed by some soothing draught, such as ammonium bromide, with aromatic spirit of ammonia, and to give from five to ten drops of solution of morphine in chloroform water, to prepare the patient by rest for the active measures which cannot be delayed. A dose of calomel is to be administered at once, and to be followed half an hour later with a senna draught. Cardiac treatment is the next consideration. Arrangements ought to be made for the immediate supply of oxygen, which is needed for continuous administration. If, asks Dr. Ewart, oxygen is worth administering in desperate states and to the dying, why should it be neglected in the early stages? Next is the question of bleeding or leeching. While not committing himself in favour of bleeding in the early stages, Dr. Ewart is quite definite on the benefits to be obtained by leeching, which he makes a routine of treatment; it relieves the pain almost invariably, and has other advantages. Other indications are to produce diaphoresis, diuresis, anti-fibrinosis, and absorption, and these ends he attempts to achieve by the following drugs: Ammonium citrate, spiritus aetheris nitrosi, iodide of potassium, and calomel, while alcohol is also useful as a cardiac stimulant. He uses the following prescription for adults:—

| | | |
|---|---|---|
| ℞ | Pot. iod. ... ... ... | gr. v. |
| | Liq. ammon. cit. ... ... | ʒ ij. |
| | Spir. aeth. nit. ... ... | ʒ ss. |
| | Spir. ammon. arom. ... | ♏ xx. |
| | Aq. chlor. ... ... ... | ad ℥ ss. |
| | Ft. mist. | |

One tablespoonful, diluted, to be taken every hour for six doses, and subsequently every three hours.

In addition, calomel is given in doses of one-sixth grain every four hours. Alcohol he regards as indispensable from the first, in moderation, as a stimulant and a food substitute. Food must be light, and small in quantity, because gastro intestinal fermentation must be absolutely avoided as one of the worst dangers; the risks of comparative starvation are as nothing in comparison. Water is a more urgent indication than food, and the more watery the diet is the more harmless and suitable will it be. Milk is too heavy and too nutritious; whey is preferable. So much for the treatment of pneumonia in the early stages. In cases which do not respond to this treatment, and which get worse, a second leeching or a venesection may be necessary, and other therapeutic measures will be indicated according to the exigencies of each particular case; but as soon as there is progression to recovery, food becomes the best and only necessary medicine.

[1] Lancet, Jan. 21, 1905.

